# Uniaxial strain control of spin-polarization in multicomponent nematic order of BaFe_2_As_2_

**DOI:** 10.1038/s41467-018-03377-8

**Published:** 2018-03-13

**Authors:** T. Kissikov, R. Sarkar, M. Lawson, B. T. Bush, E. I. Timmons, M. A. Tanatar, R. Prozorov, S. L. Bud’ko, P. C. Canfield, R. M. Fernandes, N. J. Curro

**Affiliations:** 1 Department of Physics, University of California, Davis, CA 95616 USA; 2 Institute for Solid State Physics, TU Dresden, D-01069 Dresden Germany; 3 Ames Laboratory U.S. DOE and Department of Physics and Astronomy, Iowa State University, Ames, IA 50011 USA; 4 School of Physics and Astronomy, University of Minnesota, Minneapolis, MN 55455 USA

## Abstract

The iron-based high temperature superconductors exhibit a rich phase diagram reflecting a complex interplay between spin, lattice, and orbital degrees of freedom. The nematic state observed in these compounds epitomizes this complexity, by entangling a real-space anisotropy in the spin fluctuation spectrum with ferro-orbital order and an orthorhombic lattice distortion. A subtle and less-explored facet of the interplay between these degrees of freedom arises from the sizable spin-orbit coupling present in these systems, which translates anisotropies in real space into anisotropies in spin space. We present nuclear magnetic resonance studies, which reveal that the magnetic fluctuation spectrum in the paramagnetic phase of BaFe_2_As_2_ acquires an anisotropic response in spin-space upon application of a tetragonal symmetry-breaking strain field. Our results unveil an internal spin structure of the nematic order parameter, indicating that electronic nematic materials may offer a route to magneto-mechanical control.

## Abstract (web summary)

A fundamental understanding of nematic order is one of the most important issues to explore in the high temperature superconductors. Here, the authors unveil an internal spin structure of the nematic order in BaFe_2_As_2_ by using nuclear magnetic resonance under precisely controlled tunable strain.

## Introduction

In the absence of external strain, BaFe_2_As_2_ undergoes a weakly first-order antiferromagnetic phase transition at *T*_N_ = 135 K, accompanied by an orthorhombic structural distortion that breaks the tetragonal symmetry of the unit cell in the paramagnetic phase^1–4^. The relatively small orthorhombic lattice distortion (~0.3%)^5–7^ is driven by a nematic instability^8^, whose electronic origin is manifested by the large in-plane resistivity anisotropy (~100%)^9,10^. Despite being nearly simultaneous in BaFe_2_As_2_, the nematic and antiferromagnetic transition temperatures, *T*_s_ and *T*_N_, split upon doping, giving rise to a regime with long-range nematic order but no antiferromagnetic order, since *T*_N_ < *T*_s_^1,11^.

The close relationship between nematicity and the magnetic degrees of freedom can be seen directly from the stripe-like nature of the antiferromagnetic state, which orders with one of two possible wave-vectors related by a 90° rotation: **Q**_1_ = (*π*, 0) (corresponding to spins parallel along the *y*-axis and antiparallel along *x*) and **Q**_2_ = (0, *π*) (corresponding to spins parallel along *x* and antiparallel along *y*). Below *T*_N_ nearest neighbor spins are parallel or antiparallel depending on whether they are connected by a short or long bond, however, in the nematic phase above *T*_N_ but below *T*_s_ the magnetic fluctuations centered around **Q**_1_ become weaker or stronger than those centered around **Q**_2_, depending on whether the *b*-axis is parallel or perpendicular to **Q**_1_, respectively. Mathematically, this allows one to define the nematic order parameter $\bar \varphi$ in terms of the (spin unpolarized) magnetic susceptibility *χ*(***q***) according to $\bar \varphi \equiv \chi ^{ - 1}({\bf{Q}}_2) - \chi ^{ - 1}({\mathbf{Q}}_1)$^2^. Such an interplay between nematic and spin degrees of freedom has been indeed observed by neutron scattering^6,7,12,13^ and nuclear magnetic resonance (NMR) experiments in twinned and detwinned doped BaFe_2_As_2_, LaFeAsO and NaFe_1−*x*_Co_*x*_As crystals^14–18^.

However, orbital degrees of freedom also participate actively in the nematic phase. This leads to the well-known effect that tetragonal symmetry-breaking is also manifested by a ferro-orbital polarization that makes the occupation of the Fe *d*_*xz*_ orbitals different than the occupation of the Fe *d*_*yz*_ orbitals^19^. Spin-orbit coupling (SOC), which converts anisotropies in real space into anisotropies in spin space, plays a central role controlling the interplay between spin and nematic degrees of freedom^20^. On one hand, SOC enforces the spins to point along the ordering vector direction below *T*_N_. This effect takes place even at zero applied strain, and is manifested by the fact that the three diagonal magnetic susceptibilities, *χ*_*αα*_(**Q**_1_), where *α* = *x*, *y*, *z*, are different already in the paramagnetic tetragonal phase. Indeed, the distinct behaviors of in-plane and out-of-plane spin fluctuations is well documented in the literature via polarized neutron scattering measurements^21,22^, NMR measurements^23–25^, and theoretical considerations^26^.

The evolution of the spin fluctuation anisotropy under strain has been less explored, but can shed light on the unique spin-space structure of the nematic order parameter. This is defined mathematically by $\bar \varphi _{\alpha \beta } = \chi _{\alpha \alpha }^{ - 1}({\mathbf{Q}}_2) - \chi _{\beta \beta }^{ - 1}({\mathbf{Q}}_1)$. Clearly, the nematic order parameter $\bar \varphi$ defined above can be understood as an average over all possible polarizations, $\bar \varphi = \frac{1}{9}\mathop {\sum}\limits_{\alpha \beta } {\varphi _{\alpha \beta }}$. As discussed in Supplementary Note 1, the space-group symmetry of the iron pnictides enforces many of these combinations to vanish, yielding only three non-zero-independent components: *φ*_*xy*_, *φ*_*yx*_, and *φ*_*zz*_. This important property of spin-nematicity has not been discussed previously in the literature. Experimentally, probing the spin structure of the nematicity would require polarized neutron scattering measurements in detwinned samples above the magnetic transition temperature. Polarized experiments inside the magnetically ordered phase probe a completely different type of anisotropy, related to long-range magnetic order, and not to the fluctuation spectrum^27–29^. Elucidating this hitherto unknown spin structure of the nematic order parameter is fundamental to shed light on the intricate interplay between orbital, spin, and lattice degrees of freedom, which are ultimately responsible for the superconducting instability of the system.

In this work we perform NMR spin-lattice relaxation measurements to probe the anisotropy of the spin fluctuations under fixed strain in the paramagnetic phase of BaFe_2_As_2_. The role of the applied uniaxial strain is to provide a small tetragonal symmetry-breaking field, akin to externally applied magnetic fields in ferromagnets. In contrast to previous works, here we probe the magnetic fluctuations anisotropy both in real space and in spin space—more specifically, we determine each of the nematic susceptibilities associated with the three nematic components *φ*_*xy*_, *φ*_*yx*_, and *φ*_*zz*_. Our main result is that the three nematic components respond differently to external strain, i.e., nematic order induces not only real-space anisotropy, but also affects the spin-space anisotropy. In particular, we find that the out-of-plane spin fluctuations centered at ${\mathbf{Q}}\parallel \hat a$ are more strongly enhanced by the strain, as compared to the spin fluctuations polarized along the longer in-plane axis. This raises the interesting possibility of reversing the spin polarization of the system from in-plane to out-of-plane by applying a sufficiently strong in-plane strain. More broadly, our results thus open a new avenue toward magneto-mechanical manipulation of strongly correlated systems that display nematic order.

## Results

### NMR under uniaxial strain

Key to this study is our ability to control precisely the uniaxial strain applied in the sample, which is achieved by integrating a novel piezoelectric strain cell with an NMR probe. This new device is based upon a design used previously to investigate the superconducting transition temperature of Sr_2_RuO_4_^30–32^, and can achieve both positive and negative strains with large strain homogeneity. This device differs from the horseshoe-clamp^9^ used previously for NMR^16^, and offers superior control over the sample alignment and the level of strain applied.

**Fig. 1:**
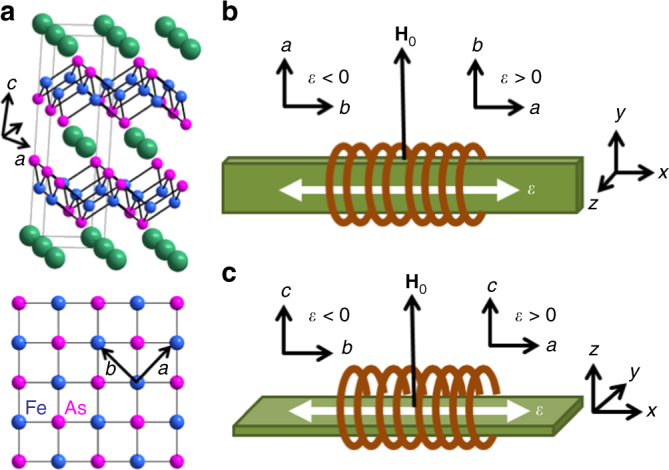
Application of uniaxial strain. **a** Crystal structure of BaFe_2_As_2_, with Ba (green), Fe (blue) and As (magenta) sites shown. Lower panel shows the Fe–As plane in the tetragonal phase, with arrows indicating the unit cell axes of the orthorhombic phase $( {{a}\parallel {(110)}_{\rm tet}},\,{b}\parallel ({{1}\mathop {1}\limits^ - {1}} )_{\rm tet} )$. **b**, **c** Orientation of the magnetic field with respect to the coil (**H**_1_) and strain axis for **H**_0_ ⊥ *c* (**b**) and **H**_0_ ∥ *c* (**c**). For positive (tensile) strain **H**_0_ is parallel to (**b**), whereas for negative (compressive) strain **H**_0_ is along (**a**)

Single crystals of BaFe_2_As_2_ were cut along the tetragonal (110) direction and mounted in the cryogenic strain cell with field oriented both parallel and perpendicular to the crystallographic *c*-axis, as shown in Fig. 1. The strain cell contains two sets of piezoelectric stacks, one inner and two outer. Because the sample is freely suspended between the piezoelectric stacks rather than glued down over a portion of the stack, the full displacement of each stack is transferred to the sample. As a result, the device is able to achieve displacements of ±6 μm at room temperature and ±3 μm at 4 K, corresponding to strains of the order of 10^−3^ in this material. A free-standing NMR coil was placed around the sample prior to securing the ends of the crystal in the strain device with epoxy. The radiofrequency field **H**_1_ is oriented parallel to the strain axis, which is always perpendicular to the external field, **H**_0_. In our device, strain is always applied along the *x*-axis defined in Fig. 1; since the *b*-axis is defined as the shorter axis, positive (i.e., tensile) strain corresponds to $x\parallel a$ and $y\parallel b$, whereas negative (i.e., compressive) strain gives $y\parallel a$ and $x\parallel b$. When the crystal is strained by applying voltage to the piezoelectric stacks, the displacement, *x*, is measured by a capacitive dilatometer, and strain is calculated as *ε* = (*x* − *x*_0_) / *L*_0_, where *L*_0_ is the unstrained length of the crystal. To account for differential thermal contraction, the zero-strain displacement, *x*_0_, was determined by the condition that the quadrupolar splitting *ν*_*αα*_ satisfies the tetragonal-symmetry relationship |*ν*_*xx*_| = |*ν*_*yy*_| = |*ν*_*zz*_| / 2, as described in Supplementary Note 2. The linear relationship between *ν*_*αα*_ and strain (Supplementary Figure 1) indicates that both positive and negative strains are achieved, without bowing of the crystal. The field **H**_0_ was oriented either along the *z*-direction parallel to the crystalline *c*-axis, or in the plane of the crystal along the *y*-direction, as shown in Fig. 1.

### Response of spin susceptibility to strain

**Fig. 2:**
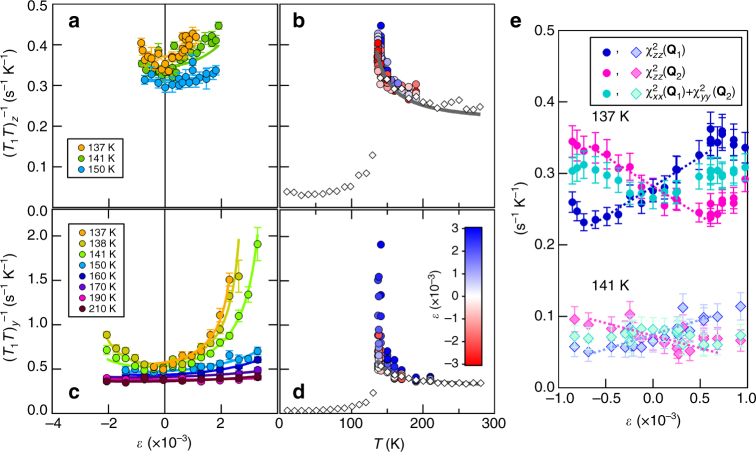
Strain and temperature dependence of the spin-lattice relaxation rate. $(T_1T)_{{\mathrm{y}},z}^{ - 1}$ vs. strain (**a**, **c**) and vs. temperature (**b**, **d**). The solid lines are fits as described in the text, and error bars are determined from least squared fitting as described under Methods. The open diamonds in **b**, **d** are reproduced from ref. ^32^. **e** *χ*_z*z*_(**Q**_1_), *χ*_z*z*_(**Q**_2_), and *χ*_x*x*_(**Q**_1_) + *χ*_y*y*_(**Q**_2_) as a function of strain at 137 K and 141 K. The data have been displaced vertically for clarity. The dashed lines are guides to the eye, and the error bars are determined by propagating the errors in **a**–**d**

The ^75^As (*I* = 3/2) spin lattice relaxation rate divided by temperature, $(T_1T)_\mu ^{ - 1}$, for different field orientations *μ* = *z*, *y* is shown in Fig. 2 both as a function of strain *ε* and temperature *T*. It is striking that while $(T_1T)_{\mathrm{z}}^{ - 1}$ increases by ~30% at 137 K for the largest applied strain (~0.3%), $(T_1T)_{\mathrm{y}}^{ - 1}$ increases by 500%. In both cases, both positive and negative strain increase (*T*_1_*T*)^−1^ in a nonlinear fashion. This behavior is a manifestation of the spin anisotropy induced by nematic order, and the enhancement of *T*_N_ under strain. More precisely, the spin lattice relaxation rate is primarily dominated by the fluctuations of the local hyperfine field at the As site, which in turn is determined by the neighboring iron spins according to:

1 $$\left( {\frac{1}{{T_1T}}} \right)_\mu = \frac{{\gamma ^2}}{2}\mathop {{{\mathrm{lim}}}}\limits_{\omega \to 0} \mathop {\sum}\limits_{{\bf{q}},\alpha ,\beta } {{\cal F}_{\alpha \beta }^{(\mu )}({\mathbf{q}})\frac{{Im\chi _{\alpha \beta }({\bf{q}},\omega )}}{{\hbar \omega }}} ,$$

where *γ* is the nuclear gyromagnetic factor, ${\cal F}_{\alpha \beta }^{(\mu )}$ are the hyperfine form factors, which depend on the field direction *μ* (Supplementary Note 3), *χ*_*αβ*_(**q**, *ω*) is the dynamical magnetic susceptibility, and *α*,*β* = {*x*, *y*, *z*}^23^. Because the system is metallic, spin fluctuations experience Landau damping, resulting in the low-energy dynamics $\chi _{\alpha \beta }^{ - 1}({\bf{q}},\omega ) = \chi _{\alpha \beta }^{ - 1}({\bf{q}}) - i\hbar \omega /\Gamma$, where *Γ* is the Landau damping, as seen by neutron scattering experiments^33^. Consequently, $\mathop {{{\mathrm{lim}}}}\limits_{\omega \to 0} \frac{{Im\chi _{\alpha \beta }({\bf{q}},\omega )}}{{\hbar \omega }} = \frac{1}{\Gamma }\chi _{\alpha \beta }^2({\bf{q}})$, i.e., the spin-lattice relaxation rate is proportional to the squared susceptibility integrated over the entire Brillouin zone.

**Fig. 3:**
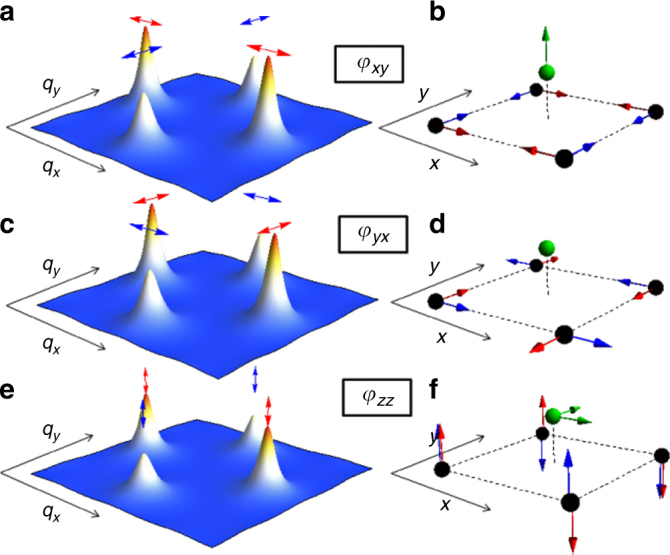
Spin-space structure of the spin-nematic order parameter. Spin fluctuations in momentum space (left) and in real space (right) and polarization directions of the Fe spins for the three nematic components, *φ*_*xy*_ (**a**, **b**), *φ*_*yx*_ (**c**, **d**), and *φ*_*zz*_ (**e**, **f**). The red arrows correspond to the magnetic ordering vector **Q**_1_ = (*π*, 0) and the blue arrows correspond to **Q**_2_ = (0, *π*). The black spheres are the Fe sites, the green sphere is the As site, and the green arrows indicate the direction of the hyperfine field

Since the magnetically ordered state has wave-vectors **Q**_1_ = (*π*, 0) and **Q**_2_ = (0, *π*), one expects that the susceptibility is peaked at these two momenta, as demonstrated in Fig. 3. Indeed, neutron scattering experiments confirm that the magnetic spectral weight is strongly peaked at **Q**_1_ and **Q**_2_^12^. A finite nematicity corresponds to a difference in the relative weights of these peaks, and the physical meaning of each component of the nematic order, *φ*_*αβ*_, is depicted in Fig. 3; for instance, *φ*_*xy*_ is a measure of the asymmetry between spin fluctuations peaked at **Q**_1_ and polarized along the *x*-axis, and spin fluctuations peaked at **Q**_2_ and polarized along the *y*-axis. The magnetic fluctuations associated with each spin polarization pattern generate very different types of fluctuating local hyperfine fields experienced by the As, which couples to the four nearest neighbor Fe spins via a transferred hyperfine interaction (Fig. 3)^23^.

As an initial step to elucidate the effect of strain on the spin-fluctuation anisotropy, we consider that the susceptibility is sharply peaked at these two magnetic ordering vectors. Evaluation of the hyperfine form factors yields the following:

2 $$\begin{array}{*{20}{c}} {\left( {T_1T} \right)_{x}^{ - 1} \propto \chi _{{x}x}^2\left( {{\bf{Q}}_1} \right) + \chi _{{y}y}^2\left( {{\bf{Q}}_2} \right) + \chi _{{z}z}^2\left( {{\bf{Q}}_2} \right)} \\ {\left( {T_1T} \right)_{y}^{ - 1} \propto \chi _{{x}x}^2\left( {{\bf{Q}}_1} \right) + \chi _{{y}y}^2\left( {{\bf{Q}}_2} \right) + \chi _{{z}z}^2\left( {{\bf{Q}}_1} \right)} \\ {\left( {T_1T} \right)_{z}^{ - 1} \propto \chi _{{z}z}^2\left( {{\bf{Q}}_1} \right) + \chi _{{z}z}^2\left( {{\bf{Q}}_2} \right)} \end{array},$$

where the prefactors are approximately the same in all equations (see Supplementary Note 3), and proportional to the off-diagonal hyperfine matrix element ${\cal F}_{{\mathrm{x}}z}$ coupling in-plane Fe spin fluctuations to out-of-plane As hyperfine fields (and vice-versa). The fact that *χ*_*zz*_(**Q**_*i*_) contributes to *T*_1_ for all directions of the applied magnetic field is thus consistent with the hyperfine field analysis depicted in Fig. 3, since out-of-plane spin fluctuations on the Fe sites produce hyperfine fluctuating fields in the As sites along both in-plane directions. Similarly, the fact that only *χ*_*xx*_(**Q**_1_) and *χ*_*yy*_(**Q**_2_) contribute to *T*_1_ for external fields applied along the plane is a consequence of the fact that these spin fluctuations generate hyperfine fields in the As site oriented out of the plane.

Because by symmetry $\left( {T_1T} \right)_{x}^{ - 1}(\varepsilon ) = \left( {T_1T} \right)_{y}^{ - 1}( - \varepsilon )$, the NMR data can be used to extract the strain and temperature dependence of the three polarized spin-susceptibility combinations $\chi _{{z}z}^2\left( {{\bf{Q}}_1} \right)$, $\chi _{{z}z}^2\left( {{\bf{Q}}_2} \right)$, and $\chi _{{x}x}^2\left( {{\bf{Q}}_1} \right) + \chi _{{y}y}^2\left( {{\bf{Q}}_2} \right)$, as shown in Fig. 2e. This analysis provides several interesting insights. First, focusing on the out-of-plane fluctuations, in-plane strain enhances spin fluctuations around one of the two ordering vectors (*χ*_*zz*_(**Q**_1_) for *ε* > 0 and *χ*_*zz*_(**Q**_2_) for *ε* < 0) at the same time as it suppresses the fluctuations around the other ordering vector. Therefore, in-plane strain transfers magnetic spectral weight between the two dominant out-of-plane spin-fluctuation channels. This is consistent with neutron scattering experiments in detwinned pnictides^6^, which, however, only probed the unpolarized susceptibility. More importantly, this behavior is a direct manifestation of the response of the nematic order parameter *φ*_*zz*_ to strain, since $\varphi _{{z}z} = \chi _{{z}z}^{ - 1}({\bf{Q}}_2) - \chi _{{z}z}^{ - 1}({\bf{Q}}_1)$.

Turning now to the average in-plane fluctuations $\chi _{{x}x}^2\left( {{\bf{Q}}_1} \right) + \chi _{{y}y}^2\left( {{\bf{Q}}_2} \right)$, we note that, in contrast to the quantity *χ*_*zz*_(**Q**_1_) − *χ*_*zz*_(**Q**_2_), it is an even function of the applied strain. This behavior can be attributed to the response of the nematic order parameter $\varphi _{{x}y} = \chi _{{x}x}^{ - 1}({\bf{Q}}_2) - \chi _{{y}y}^{ - 1}({\bf{Q}}_1)$ to strain. Similarly to *φ*_*zz*_, *φ*_*xy*_ promotes a transfer of magnetic spectral weight, but now between *x*-polarized spin fluctuations around **Q**_1_ and *y*-polarized spin fluctuations around **Q**_2_. Since only the combination $\chi _{{x}x}^2\left( {{\bf{Q}}_1} \right) + \chi _{{y}y}^2\left( {{\bf{Q}}_2} \right)$ contributes to the spin-lattice relaxation rate, the total magnetic spectral weight remains the same to linear order in *φ*_*xy*_, since what is suppressed in, say, *χ*_*yy*_(**Q**_2_) is tranferred to *χ*_*xx*_(**Q**_1_). Of course, as strain is enhanced, nonlinear effects quadratic in $\varphi _{{\mathrm{x}}y}^2$ take place, in agreement with the behavior displayed by Fig. 2e. Note that the third nematic order parameter, $\varphi _{{y}x} = \chi _{{y}y}^{ - 1}({\bf{Q}}_2) - \chi _{{x}x}^{ - 1}({\bf{Q}}_1)$, does not affect the in-plane fluctuations that contribute the most to the spin-lattice relaxation rate. This is not unexpected, since the spin fluctuations associated with *χ*_*yy*_(**Q**_1_) and *χ*_*xx*_(**Q**_2_) do not generate hyperfine fields in the As sites, as shown in Fig. 3.

The most striking feature of Fig. 2e is that the out-of-plane spin fluctuations seem to have a larger response to in-plane strain than the in-plane spin fluctuations. This observation suggests that the nematic susceptibility associated with *φ*_*zz*_, $\chi _{{zz}}^{{\mathrm{nem}}} \equiv \partial \varphi _{{z}z}/\partial \varepsilon$, is larger than the nematic susceptibility associated with *φ*_*xy*_, $\chi _{{xy}}^{{\mathrm{nem}}} \equiv \partial \varphi _{{xy}}/\partial \varepsilon$, and is manifestation of the fact that nematic order induces not only real-space anisotropy, but also spin-space anisotropy. To make this analysis more quantitative, we fit the full temperature, strain, and field orientation dependence of *T*_1_ to a model that incorporates the fact that the magnetic fluctuations are not infinitely peaked at the ordering vectors **Q**_1,2_, since the magnetic correlation length is finite above the magnetic transition. In the tetragonal phase, there are three different magnetic correlation lengths, *ξ*_*x*_, *ξ*_*y*_, and *ξ*_*z*_, associated, respectively, with the pairs of peaks (*χ*_*xx*_(**Q**_1_), *χ*_*yy*_(**Q**_2_)); (*χ*_*yy*_(**Q**_1_), *χ*_*xx*_(**Q**_2_)), and (*χ*_*zz*_(**Q**_1_), *χ*_*zz*_(**Q**_2_)). This spin anisotropy is intrinsic to the tetragonal crystalline symmetry and is enforced by the spin-orbit coupling even in the absence of nematic order as shown previously by polarized neutron scattering^21,22,27–29^. Nematic order induced by strain breaks the equivalence between these pairs of peaks, splitting the correlation lengths into $\tilde \xi _{x}^{ - 2} = \xi _{x}^{ - 2} \mp \varphi _{{x}y}$, $\tilde \xi _{y}^{ - 2} = \xi _{y}^{ - 2} \mp \varphi _{{y}x}$, and $\tilde \xi _{z}^{ - 2} = \xi _{z}^{ - 2} \mp \varphi _{{z}z}$. This model is similar to the one used previously in ref. ^16^ and is described in Supplementary Note 3.

**Fig. 4:**
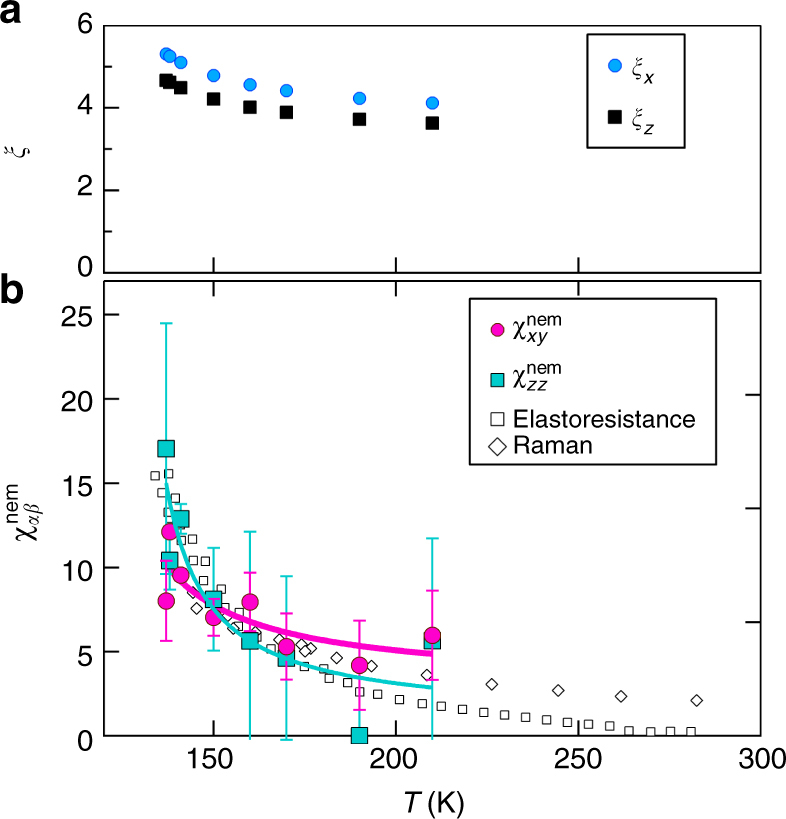
Temperature dependence of the nematic susceptibilities. **a** Correlation lengths *ξ*_*x,z*_(0) at zero strain, and **b** nematic susceptibilities $\chi _{{xy,zz}}^{{\mathrm{nem}}}$ vs. temperature, based on the fits (solid lines) shown in Fig. 2. Also shown are the nematic susceptibilities measured by Raman and elastoresistance measurements, reproduced from refs. ^35–37^, respectively. The solid lines are fits as described in the text. The error bars are determined from least squared minimization fits, holding the *ξ*_*x,z*_(0) parameters fixed, as described in Supplementary Note 4

The fits for $(T_1T)_{z}^{ - 1}$ and $(T_1T)_{y}^{ - 1}$ in the absence of strain are shown as solid gray lines in Fig. 2b, d for *ξ*_*x*_ = *ξ*_*y*_. We find *ξ*_*z*_ / *ξ*_*x*_ = 0.88, in agreement with the fact that in the absence of strain the spins point along the plane. Moreover, the temperature dependence of *ξ*_*x*_(*T*), shown in Fig. 4a, gives values consistent with those measured by inelastic neutron scattering^34^. Having fixed the unstrained parameters, we perform fits in the presence of strain, shown by the solid lines in Fig. 2a, c. The only parameters introduced in this case are *φ*_*xy*_ and *φ*_*zz*_. The good agreement between the fitted and the experimental curves of both $(T_1T)_{z}^{ - 1}$ and $(T_1T)_{y}^{ - 1}$ over a wide temperature-strain regime demonstrates the suitability of the phenomenological model employed in our analysis.

The temperature and strain behaviors of the nematic order parameters *φ*_*αβ*_ allows us to extract the temperature dependence of the nematic susceptibilities $\chi _{{xy}}^{{\mathrm{nem}}}$ and $\chi _{{zz}}^{{\mathrm{nem}}}$, as shown in Fig. 4b. The data suggest that $\chi _{{zz}}^{{\mathrm{nem}}} > \chi _{{xy}}^{{\mathrm{nem}}}$, particularly close to the magnetic transition. This quantitative analysis corroborates the qualitative conclusion above, namely that nematic order induces anisotropies in spin-space, and that the out-of-plane spin fluctuations are more strongly enhanced by in-plane strain than the in-plane spin fluctuations. The in-plane spin fluctuations, are nevertheless larger, giving rise to in-plane ordering at *T*_N_.

It is interesting to compare $\chi _{{xy}}^{{\mathrm{nem}}}$ and $\chi _{{zz}}^{{\mathrm{nem}}}$ with the nematic susceptibility extracted from elastoresistance^37^ and from electronic Raman spectroscopy experiments^35^. As shown in Fig. 4b, the values are consistent, and the NMR-extracted nematic susceptibilities also follow a Curie–Weiss type of behavior^36^, with a Curie temperature *T*_0_ = 116 K comparable to that extracted from the elastoresistance^37^. Note, however, that, in contrast to our NMR analysis, the other probes for the nematic susceptibility are not sensitive to the spin-space structure of the nematic susceptibility.

## Discussion

To the best of our knowledge, our results are the first to reveal the internal spin structure of the nematic order parameter in iron-based superconductors. This behavior is a clear manifestation of the entanglement between spin, orbital, and lattice degrees of freedom in the normal state of these compounds. Since superconductivity emerges from this unique state, the rich interplay between these different degrees of freedom revealed by our NMR analysis will certainly affect the properties of the superconducting state.

The surprising anisotropic response of different nematic components to in-plane strain reveals that the spin polarization can be controlled by lattice distortions, similar to a piezomagnetic effect. In particular, the result $\chi _{{z}z}^{{\mathrm{nem}}} > \chi _{{x}y}^{{\mathrm{nem}}}$ implies that for sufficiently large strain ε*, the dominant spin polarization will shift from in-plane to out-of-plane. Recent NMR and neutron studies in unstrained FeSe have uncovered similar evidence for a large spin susceptibility along the *c*-axis in the nematic phase, above *T*_*c*_^38,39^. However, the observation of large *c*-axis spin fluctuations in FeSe does not reveal information about the temperature dependence of the various nematic susceptibility components, $\chi _{\alpha \beta }^{{\mathrm{nem}}}$, which necessarily require the application of strain. For BaFe_2_As_2_, the value of ε* can be estimated from the condition that the out-of-plane magnetic correlation length $\tilde \xi _{z}^{ - 2} = \xi _{z}^{ - 2} - \chi _{{zz}}^{{\mathrm{nem}}}\varepsilon$ becomes larger than the in-plane magnetic correlation length $\tilde \xi _{x} = \xi _{x} - \chi _{{xy}}^{{\mathrm{nem}}}\varepsilon$, yielding ε* ≈ 0.4% close to the magnetic transition temperature, assuming a linear strain response. Such a strain value, which is just beyond the capability of our specific piezo device, can reasonably be achieved by similar types of devices, however. More importantly, this analysis opens a new avenue to control spin polarization in nematic materials without using magnetic fields, but instead by using mechanical strain. Since nematic order has been observed in other correlated materials such as cuprates and ruthenates, it will be interesting to investigate whether similar sizable effects are present in these systems as well.

More broadly, our work demonstrates that precision tunable strain in combination with NMR provides a novel and important method to probe spin and charge degrees of freedom. It provides an intriguing possibility to tune the NMR spin relaxation rate by changing a voltage bias on the piezoelectric stacks. The subtle coupling between the lattice and spin polarizations exhibited by BaFe_2_As_2_ offers the potential for controlling magnetic properties through lattice deformations in next-generation materials. Another potential application of our technique is the use of nuclear quadrupolar resonance to image local strains. The large response of the EFG to strain observed in this study would translate into high spatial resolution in a linear strain gradient, so that As NMR may be able to resolve microscopic features such as grain boundaries or defects.

## Methods

### Sample mounting

Crystals were grown in self-flux as described in ref. ^40^ and in Supplementary Note 5, and cut along the (110)_T_ direction. Sample A had a mass of 2.52 mg and was mounted with the field parallel to the *c*-axis, and Sample B had a mass 0.91 mg and was mounted with the field perpendicular to the *c*-axis (Fig. 1). The crystals were secured with heat-cured epoxy (UHU Plus 300 epoxy resin). Strain was applied along the (110)_T_ direction using the CS100 cryogenic uniaxial strain cell developed by Razorbill Instruments based on a design by Hicks et. al.^30^, mounted in a modified probe operating in a Quantum Design PPMS cryostat.

### Strain calibration

The displacement, *x*, was measured by monitoring the capacitance of using a precision capacitance bridge with a resolution of 0.1 nm. The strain was computed as *ε* = (*x* − *x*_0_) / *L*_0_, where *L*_0_ = 2.052 mm and *x*_0_ = 49.5 μm for sample A and *L*_0_ = 1.494 mm and *x*_0_ = 51.58 μm for sample B. For sample B, positive (tensile) strain corresponds to ${\bf{H}}_0 || \hat b$ and negative (compressive) strain corresponds to ${\bf{H}}_0 || \hat a$. Because the sample was mounted at room temperature, thermal contraction creates positive strain even at zero piezo bias at low temperatures, making a precise determination of *x*_0_ difficult. For sample A *x*_0_ was determined by the minimum in (*T*_1_*T*)^−1^ vs. *x*, and for sample B *x*_0_ was determined by the value *ν*_*bb*_(*x*_0_) = |*ν*_*cc*_| / 2 = 1.23 MHz, where *ν*_*αα*_ is the quadrupolar splitting for field along the *α*-direction (see Supplementary Note 2). The maximum/minimum possible applied voltages to the piezoelectric stacks limited the range of strains that could be applied to between approximately −0.002 to +0.003 in the perpendicular case, and −0.0015 to +0.002 for the parallel case.

### Spin-lattice relaxation measurements

The spin-lattice relaxation rate was measured using inversion recovery at the central transition in fixed field, and the data were fit to the expression $M(t) = M_0\left[ {1 - 2f\left( {\frac{9}{{10}}e^{ - 6t/T_1} + \frac{1}{{10}}e^{ - t/T_1}} \right)} \right]$. The data were well-fit to a single *T*_1_ value.

### Data availability

All data needed to evaluate the conclusions are present in the paper and/or supplemental materials. Correspondence and requests for materials should be addressed to N.J.C.

## Electronic supplementary material

Supplementary Information
