# Supplementary material for: Uniaxial strain control of spin-polarization in multicomponent nematic order of BaFe2As2
Source: Nat Commun. 2018 Mar 13;9:1058. doi: 10.1038/s41467-018-03377-8 (PMC5849640; doi:10.1038/s41467-018-03377-8)
Supplement: Supplementary file 1 — Supplementary Information [file 41467_2018_3377_MOESM1_ESM.pdf]

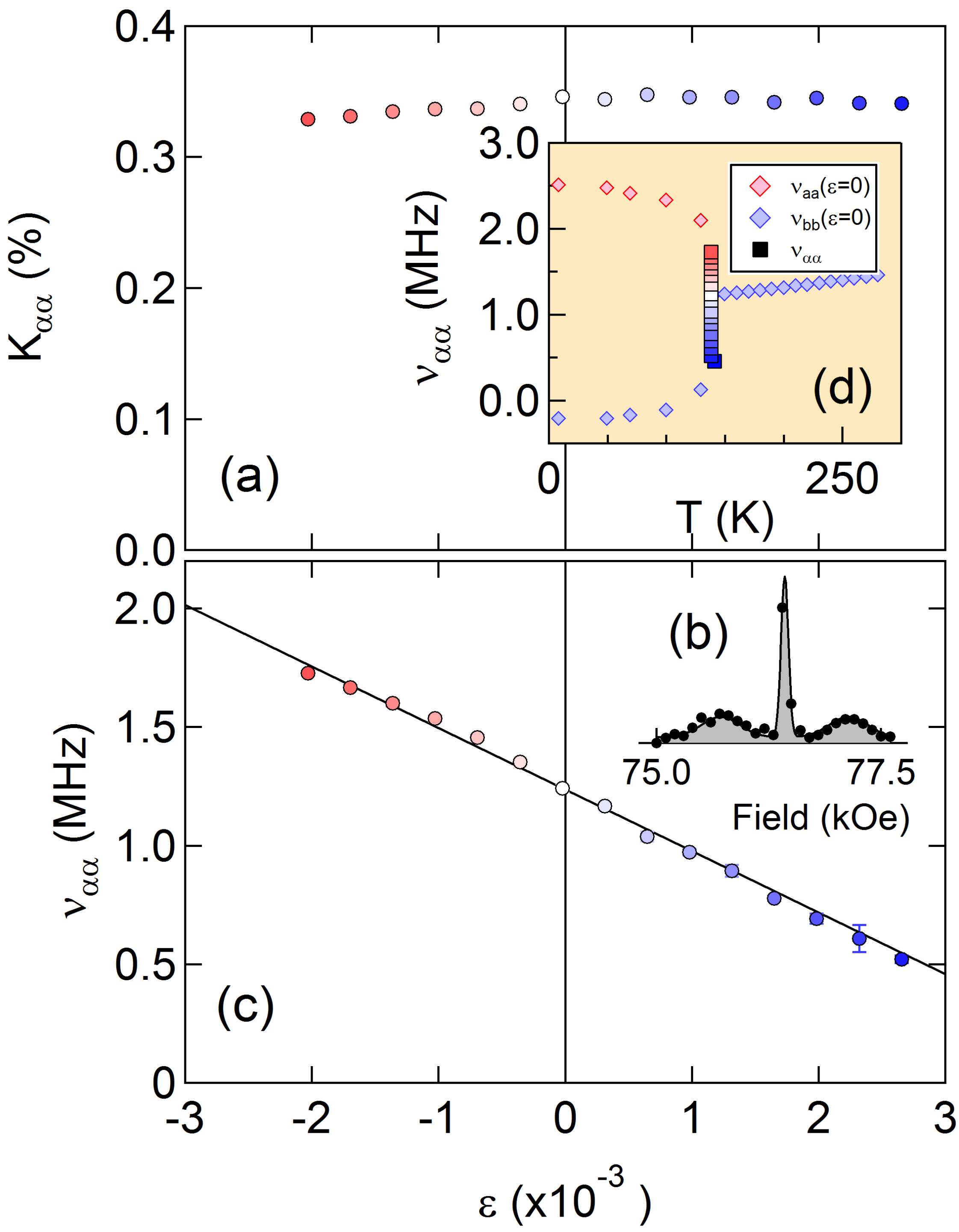

Supplementary Figure 1. **Knight shift and EFG Versus Strain.** (a) Knight shift versus strain at 138K. (b) The ^{75}As spectrum at 138K for a strain level of 0.0265% at frequency 55.924 MHz. The solid line is a fit to the spectrum as described in the text. (c) The quadrupolar splitting versus strain, and (d) versus temperature. The zero-strain points (diamonds) are reproduced from Ref. 1.

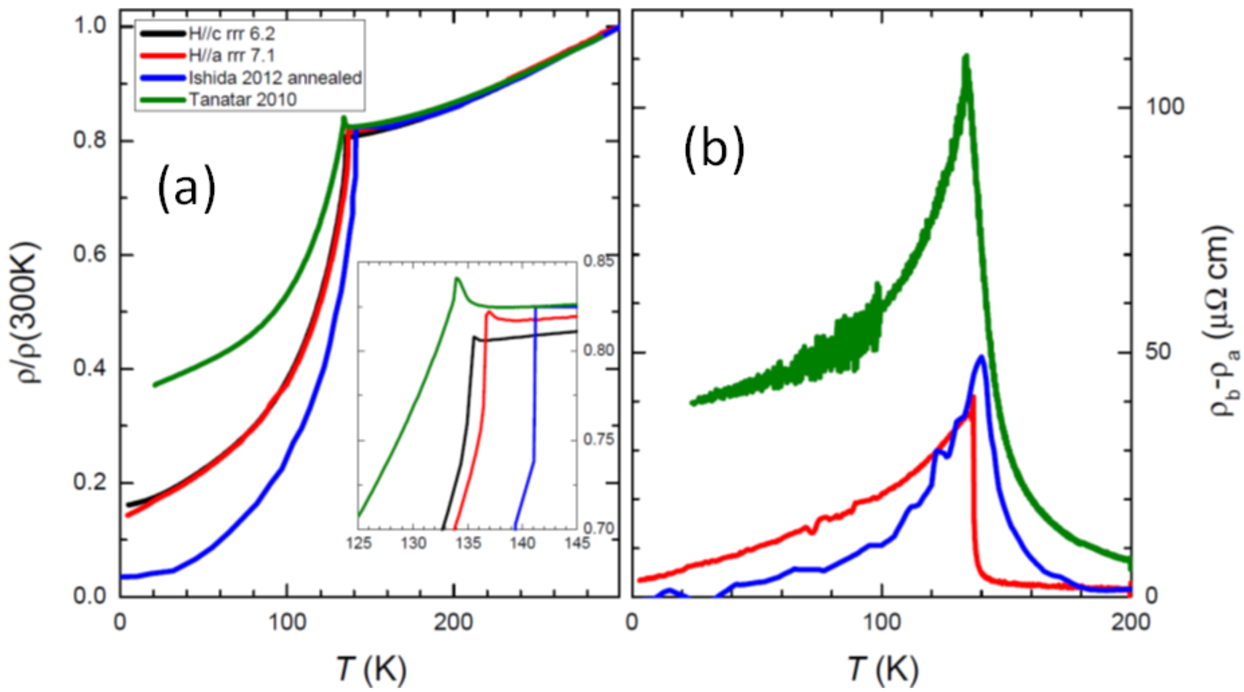

Supplementary Figure 2. Resistivity Versus Temperature of Measured Samples. (a) Temperature-dependent electrical resistivity $\rho(T)$, of the samples used in this work for $H \parallel a$ and $H \parallel c$ configurations, shown using a normalized $\rho/\rho(300K)$ scale. For reference we show data on as-grown samples (Green, Tanatar *et al.* [2]), and on annealed samples (Blue, Ishida *et al.* [3]). The inset shows the same data focusing on the structural/magnetic transition, revealing a systematic shift of the T_s feature to higher temperatures with increase of residual resistivity ratio. (b) The in-plane resistivity difference $\rho_b - \rho_a$ in the orthorhombic phase for the same samples.

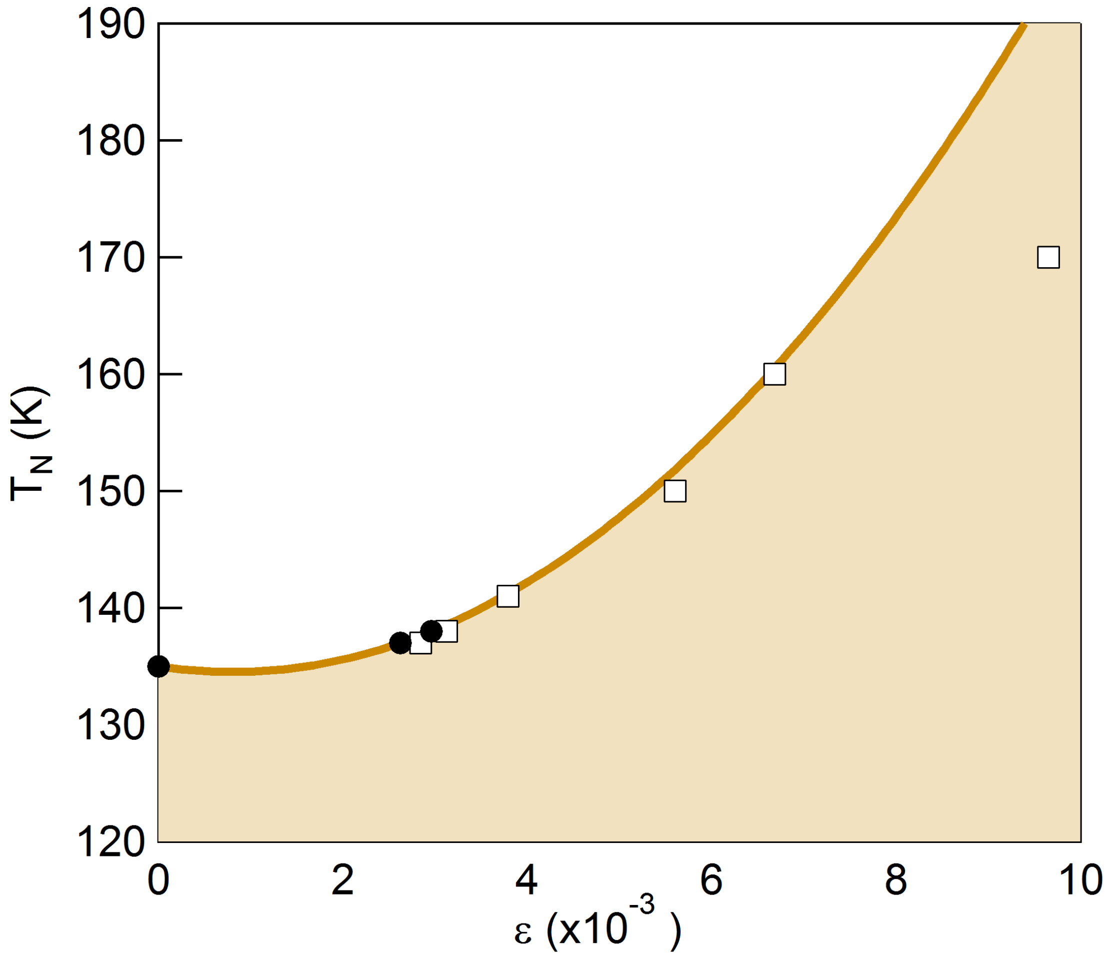

Supplementary Figure 3. T_{N} **Versus Strain**. T_{N} versus strain measured experimentally (solid black circles) and based on the measured fit parameters for ξ_a and $\chi_{\mathrm{xy}}^{\mathrm{nem}}$ (open squares). The solid line is a guide to the eye.

Supplementary Note 1. SPIN-SPACE STRUCTURE OF THE NEMATIC ORDER PARAMETER

The nematic order parameter naturally acquires an internal spin structure, since generically one must define $\varphi_{\alpha\beta} = \chi^{-1}_{\alpha\alpha}(\mathbf{Q}_2) - \chi^{-1}_{\beta\beta}(\mathbf{Q}_1)$. The space-group symmetry of the iron pnictides enforces many of these combinations to vanish, yielding only three non-zero independent components: φ_{xy}, φ_{yx}, and φ_{zz}. To make these points sharper, we employ simple Ginzburg-Landau considerations. There are two magnetic order parameters, $\mathbf{M}_1$ and $\mathbf{M}_2$, which correspond to the propagation vectors $\mathbf{Q}_1 = (\pi, 0)$ (stripes of anti-parallel spins along

the x axis) and $\mathbf{Q}_2 = (0, \pi)$ (stripes of anti-parallel spins along the y axis), respectively. These are Heisenberg spins, thus the vectors have 3 components. If the spins were completely uncoupled to the lattice, the free energy F would be isotropic in spin space, and therefore would depend only on $M_1^2 = M_{1,\mathrm{x}}^2 + M_{1,\mathrm{y}}^2 + M_{1,\mathrm{z}}^2$ and $M_2^2 = M_{2,\mathrm{x}}^2 + M_{2,\mathrm{y}}^2 + M_{2,\mathrm{z}}^2$. Because M_1 and M_2 are related by a 90° rotation and because the system is tetragonal, the quadratic term of the free energy can only depend on the combination $(M_1^2 + M_2^2)$, i.e. $F = a\,(M_1^2 + M_2^2) + \mathcal{O}\,(M^4)$, where $a \propto T - T_{\mathrm{N}}$, and T_{N} is the mean-field transition temperature. Note that, here, below T_{N}, the magnetization can point in any direction in spin space.

Now, in the real systems, the spins are coupled to the crystal fields via the spin-orbit coupling (SOC). This induces spin anisotropies that enforce the spins to point along certain directions. As demonstrated in Ref. [4], in a tetragonal system (i.e. above T_{N} and the nematic/structural transition T_{s}), there are three spin-anisotropic terms generated by the SOC:

$$F = a\left(M_1^2 + M_2^2\right) + \alpha_1\left(M_{1,\mathrm{x}}^2 + M_{2,\mathrm{y}}^2\right) + \alpha_2\left(M_{1,\mathrm{y}}^2 + M_{2,\mathrm{x}}^2\right) + \alpha_3\left(M_{1,\mathrm{z}}^2 + M_{2,\mathrm{z}}^2\right) + \mathcal{O}\left(M^4\right) \quad (1)$$

The first additional term corresponds to $\mathbf{M}_i \parallel \mathbf{Q}_i$, the second additional term corresponds to $\mathbf{M}_i \perp \mathbf{Q}_i$ and in-plane, and the third additional term corresponds to $\mathbf{M}_i \perp \mathbf{Q}_i$ and out-of-plane. Clearly, the smaller α_i determines the direction of the magnetization below the magnetic transition temperature. Importantly, note that tetragonal symmetry is fully preserved: spin fluctuations polarized along $x/y/z$ and centered at $\mathbf{Q}_1$ are equivalent to spin fluctuations polarized along $y/x/z$ (respectively) and centered at $\mathbf{Q}_2$. Polarized inelastic neutron scattering or NMR spin-lattice relaxation rate measurements in the paramagnetic phase probe precisely these terms. This has been widely explored in the literature.

Nematic order is manifested in the spin spectrum as an inequivalence between magnetic fluctuations centered around $\mathbf{Q}_1$ and $\mathbf{Q}_2$. If the system had full spin-rotational invariance, the spin-nematic order parameter would be then simply:

$$\varphi = \left\langle M_1^2 \right\rangle - \left\langle M_2^2 \right\rangle$$

This is precisely the order parameter that has been widely explored in the theoretical literature, and measured experimentally in unpolarized inelastic neutron scattering mea-

surements in detwinned samples. However, because the SOC introduces spin anisotropies, there are actually different combinations of spin-polarized fluctuations centered around $\mathbf{Q}_1$ and $\mathbf{Q}_2$. They can be derived from symmetry considerations: as shown in the free energy (1), tetragonal symmetry imposes that spin fluctuations polarized along $x/y/z$ and centered at $\mathbf{Q}_1$ are equivalent to spin fluctuations polarized along $y/x/z$ (respectively) and centered at $\mathbf{Q}_2$. Thus, there are three different combinations that break the equivalence between fluctuations centered around $\mathbf{Q}_1$ and $\mathbf{Q}_2$:

$$\begin{aligned}\varphi_{\mathrm{xy}} &= \left\langle M_{1,\mathrm{x}}^2 \right\rangle - \left\langle M_{2,\mathrm{y}}^2 \right\rangle \\ \varphi_{\mathrm{yx}} &= \left\langle M_{1,\mathrm{y}}^2 \right\rangle - \left\langle M_{2,\mathrm{x}}^2 \right\rangle \\ \varphi_{\mathrm{zz}} &= \left\langle M_{1,\mathrm{z}}^2 \right\rangle - \left\langle M_{2,\mathrm{z}}^2 \right\rangle \end{aligned} \tag{2}$$

The fact that these three terms break the same tetragonal symmetry implies that they must be either all zero or all non-zero. However, the SOC enforces different values for these three order parameters. In other words, the nematic order parameter naturally acquires a spin structure due to the SOC, i.e. the real-space anisotropy becomes entangled with the spin-space anisotropy.

The uniaxial strain ε applied to the system breaks explicitly the tetragonal symmetry and induces an additional contribution to the free energy:

$$\delta F = \lambda_1 \varepsilon \left(M_{1,\mathrm{x}}^2 - M_{2,\mathrm{y}}^2 \right) + \lambda_2 \varepsilon \left(M_{1,\mathrm{y}}^2 - M_{2,\mathrm{x}}^2 \right) + \lambda_3 \varepsilon \left(M_{1,\mathrm{z}}^2 - M_{2,\mathrm{z}}^2 \right)$$

By varying the external strain ε, we can then extract the nematic susceptibility of each spin-nematic order parameter separately:

$$\chi_{\mathrm{xy}}^{\mathrm{nem}} = \frac{\partial \varphi_{\mathrm{xy}}}{\partial \varepsilon} \quad , \quad \chi_{\mathrm{yx}}^{\mathrm{nem}} = \frac{\partial \varphi_{\mathrm{yx}}}{\partial \varepsilon} \quad , \quad \chi_{\mathrm{zz}}^{\mathrm{nem}} = \frac{\partial \varphi_{\mathrm{zz}}}{\partial \varepsilon}$$

Supplementary Note 2. SPECTRAL MEASUREMENTS

When the crystal is strained by applying voltage to the piezoelectric stacks, the displacement, x, is measured by a capacitive position sensor, and strain is calculated as $\varepsilon = (x - x_0)/L_0$, where L_0 is the unstrained length of the crystal. It is crucial to determine

the unstrained displacement, x_0, at cryogenic temperatures due to differential thermal contraction between the strain device and the sample. This value can be obtained by observing the asymmetry of the electric field gradient (EFG) tensor. The spectra were measured by acquiring echoes while sweeping the magnetic field H_0 at fixed frequency. The quadrupolar satellite resonances occur at fields $H_{sat} = (f_0 \pm \nu_{\alpha\alpha})/\gamma(1 + K_{\alpha\alpha})$, where f_0 is the radiofrequency, $\gamma = 7.29019$ MHz/T is the gyromagnetic ratio, $K_{\alpha\alpha}$ and $\nu_{\alpha\alpha}$ are the Knight shift and EFG tensor components in the $\alpha = (x, y, z)$ direction. The central transition field is given by: $H_{cen} = \frac{f_0}{\gamma(1+K_{\alpha\alpha})}\left(\frac{1}{2} + \sqrt{\frac{3f_0^2-2(\nu_{\beta\beta}+\nu_{\alpha\alpha})^2}{12}}\right)$, where $\beta = (y, x, z)$ for $\alpha = x, y$, z. The center of gravity of each peak was used to determine the resonance field, and hence $K_{\alpha\alpha}$ and $\nu_{\alpha\alpha}$ as a function of strain.

Supplementary Figure 1(b) shows a typical field-swept NMR spectrum of the ^{75}As, revealing a narrow central transition ($I_z = 1/2 \leftrightarrow -1/2$) and two quadrupolar satellite peaks ($\pm 3/2 \leftrightarrow \pm 1/2$). The spectrum was fit to the sum of three Gaussians to extract both the Knight shift, $K_{\alpha\alpha}$, and the EFG, $\nu_{\alpha\alpha}$. The EFG tensor is given by $\nu_{\alpha\beta} = (eQ/12h)\partial^2 V/\partial x_\alpha \partial x_\beta$, where Q is the quadrupolar moment of the ^{75}As and V is the electrostatic potential at the As site. This quantity is dominated by the occupation of the $d_{\rm xz,yz}$ orbitals of the neighboring Fe atoms, and the EFG asymmetry $\eta = (\nu_{\rm yy}-\nu_{\rm xx})/(\nu_{\rm xx}+\nu_{\rm yy})$ is a measure of the nematic order parameter [5, 6]. Note that the magnetic field lies along the shorter b-axis under tensile strain ($\varepsilon > 0$), and along the longer a-axis under compressive strain ($\varepsilon < 0$), as shown in Supplementary Figure 2 of the main text. The EFG enables us to identify the zero-strain displacement, x_0, by the condition $|\nu_{\rm xx}| = |\nu_{\rm yy}| = |\nu_{\rm zz}|/2$. As shown in Supplementary Figure 1(c), $\nu_{\rm yy}$, and hence $\eta(\varepsilon) = (\nu_{\rm yy}(\varepsilon) - \nu_{\rm yy}(-\varepsilon))/(\nu_{\rm yy}(\varepsilon) + \nu_{\rm yy}(-\varepsilon))$, varies linearly with strain.

Despite the fact that the EFG varies with strain, we find no significant variation of the satellite linewidth with strain. The strong variation of the EFG with strain explains the quadrupolar broadening observed in Co, Ni or Cu-doped $\mathrm{Ba(Fe,M)_2As_2}$ [7–9]. The dopant atoms create an inhomogeneous strain field that gives rise to a distribution of local EFGs. Recently a finite value of $\eta \sim 0.1$ was reported in the tetragonal phase of unstrained $\mathrm{BaFe_2(As_{1-x}P_x)_2}$ above $T_{\rm s}$ [6]. The origin of this finite nematicity is likely due to local defects, and based on our results the strain fields are on the order of 0.05%.

The Knight shift is shown versus strain in Supplementary Figure 1(a) for $\mathbf{H}_0 \perp c$. The in-plane Knight shift shows little or no variation with ε, such that $(K_{\rm xx} - K_{\rm yy})/K_{\rm yy} \leq 3\%$

at the highest strain levels in this material. This result is surprising because the same quantity is approximately 6% in the nematic phase of FeSe [10]. Recent static susceptibility measurements in $BaFe_2As_2$ under strain indicate that $\chi_{\rm xx}$ and $\chi_{\rm yy}$ in the paramagnetic phase differ by only 5% [11]. This result suggests that $\chi_{\alpha\alpha}(\mathbf{q}=0)$ couples only weakly to the strain.

Supplementary Note 3. SPIN-LATTICE RELAXATION RATE MODEL

As stated in the main text, the spin-lattice relaxation rate is given by:

$$\left(\frac{1}{T_1 T}\right)_\mu = \frac{\gamma^2}{2}\sum_{\mathbf{q},\alpha,\beta}\mathcal{F}^{(\mu)}_{\alpha\beta}(\mathbf{q})\frac{\mathrm{Im}\chi_{\alpha\beta}(\mathbf{q},\omega)}{\hbar\omega} \tag{3}$$

where γ is the gyromagnetic ratio of the nuclear spin, and $\mathcal{F}^{(\mu)}_{\alpha\beta}$ is a form factor that depends on the direction of the applied field (indicated by μ), and $\alpha,\beta=\{x,y,z\}$. The coordinate system is defined such that x and y connect nearest neighbor Fe atoms. Ref. [12] derived the form factor for an As nucleus subject to an arbitrary field direction. In the paramagnetic state, one obtains (see also Ref. [13]):

$$\left(\frac{1}{T_1 T}\right)_\mu = \frac{\gamma^2}{2}\sum_{\mathbf{q}}\sum_{\alpha=1,2}\left[\bar{R}^{(\mu)}\cdot\bar{A}_{\mathbf{q}}\cdot\bar{\tilde{\chi}}(\mathbf{q})\cdot\bar{A}^{\dagger}_{\mathbf{q}}\cdot\left(\bar{R}^{(\mu)}\right)^{\dagger}\right]_{\alpha\alpha} \tag{4}$$

All quantities with an overbar are 3×3 matrices. The matrix $\bar{\tilde{\chi}}(\mathbf{q})$ is diagonal; its matrix elements are related to the magnetic susceptibility elements according to:

$$\tilde{\chi}_{\alpha\alpha}(\mathbf{q}) \equiv \lim_{\omega\to 0}\frac{\mathrm{Im}\chi_{\alpha\alpha}(\mathbf{q},\omega)}{\hbar\omega} = \frac{1}{\Gamma}\chi^2_{\alpha\alpha}(\mathbf{q}) \tag{5}$$

where Γ is the Landau damping term. Furthermore, we have the hyperfine tensor:

$$\bar{A}_{\mathbf{q}} = 4\begin{pmatrix} A_{\rm xx}\cos\left(\frac{q_{\rm x}}{2}\right)\cos\left(\frac{q_{\rm y}}{2}\right) & -A_{\rm xy}\sin\left(\frac{q_{\rm x}}{2}\right)\sin\left(\frac{q_{\rm y}}{2}\right) & iA_{\rm xz}\sin\left(\frac{q_{\rm x}}{2}\right)\cos\left(\frac{q_{\rm y}}{2}\right) \\ -A_{\rm yx}\sin\left(\frac{q_{\rm x}}{2}\right)\sin\left(\frac{q_{\rm y}}{2}\right) & A_{\rm yy}\cos\left(\frac{q_{\rm x}}{2}\right)\cos\left(\frac{q_{\rm y}}{2}\right) & iA_{\rm yz}\cos\left(\frac{q_{\rm x}}{2}\right)\sin\left(\frac{q_{\rm y}}{2}\right) \\ iA_{\rm zx}\sin\left(\frac{q_{\rm x}}{2}\right)\cos\left(\frac{q_{\rm y}}{2}\right) & iA_{\rm zy}\cos\left(\frac{q_{\rm x}}{2}\right)\sin\left(\frac{q_{\rm y}}{2}\right) & A_{\rm zz}\cos\left(\frac{q_{\rm x}}{2}\right)\cos\left(\frac{q_{\rm y}}{2}\right)\end{pmatrix} \tag{6}$$

and the rotation matrix:

$$\bar{R}^{(\mu)} = \begin{pmatrix} \sin^2\phi + \cos\theta\,\cos^2\phi & -\sin 2\phi\,\sin^2\frac{\theta}{2} & \cos\phi\,\sin\theta \\ -\sin 2\phi\,\sin^2\frac{\theta}{2} & \cos^2\phi + \cos\theta\,\sin^2\phi & \sin\phi\,\sin\theta \\ -\cos\phi\,\sin\theta & -\sin\phi\,\sin\theta & \cos\theta \end{pmatrix} \tag{7}$$

Here, the field direction μ is described by the angles θ, φ according to $\hat{\mathbf{h}} = \cos\varphi \sin\theta\, \hat{\mathbf{x}} + \sin\varphi \sin\theta\, \hat{\mathbf{y}} + \cos\theta\, \hat{\mathbf{z}}$. Because the lattice distortion is very small, we consider hereafter that the hyperfine tensor remains essentially tetragonal, i.e. $A_{\mathrm{xx}} = A_{\mathrm{yy}}$, $A_{\mathrm{yz}} = A_{\mathrm{xz}}$, and $A_{\mathrm{xy}} = A_{\mathrm{yx}}$.

It is now straightforward to obtain the expressions for $1/\left(T_1 T\right)_\mu$ for different field directions μ. We find:

$$\begin{aligned}\left(\frac{1}{T_1 T}\right)_{\mathrm{x}} = & 8\gamma^2 \sum_{\mathbf{q}} \left[\sin^2\left(\frac{q_{\mathrm{x}}}{2}\right)\sin^2\left(\frac{q_{\mathrm{y}}}{2}\right) A_{\mathrm{xy}}^2 + \sin^2\left(\frac{q_{\mathrm{x}}}{2}\right)\cos^2\left(\frac{q_{\mathrm{y}}}{2}\right) A_{\mathrm{xz}}^2\right] \tilde{\chi}_{\mathrm{xx}}(\mathbf{q}) \\ & 8\gamma^2 \sum_{\mathbf{q}} \left[\cos^2\left(\frac{q_{\mathrm{x}}}{2}\right)\cos^2\left(\frac{q_{\mathrm{y}}}{2}\right) A_{\mathrm{yy}}^2 + \cos^2\left(\frac{q_{\mathrm{x}}}{2}\right)\sin^2\left(\frac{q_{\mathrm{y}}}{2}\right) A_{\mathrm{yz}}^2\right] \tilde{\chi}_{\mathrm{yy}}(\mathbf{q}) \\ & 8\gamma^2 \sum_{\mathbf{q}} \left[\cos^2\left(\frac{q_{\mathrm{x}}}{2}\right)\sin^2\left(\frac{q_{\mathrm{y}}}{2}\right) A_{\mathrm{yz}}^2 + \cos^2\left(\frac{q_{\mathrm{x}}}{2}\right)\cos^2\left(\frac{q_{\mathrm{y}}}{2}\right) A_{\mathrm{zz}}^2\right] \tilde{\chi}_{\mathrm{zz}}(\mathbf{q}) \end{aligned} \tag{8}$$

$$\begin{aligned}\left(\frac{1}{T_1 T}\right)_{\mathrm{y}} = & 8\gamma^2 \sum_{\mathbf{q}} \left[\cos^2\left(\frac{q_{\mathrm{x}}}{2}\right)\cos^2\left(\frac{q_{\mathrm{y}}}{2}\right) A_{\mathrm{xx}}^2 + \sin^2\left(\frac{q_{\mathrm{x}}}{2}\right)\cos^2\left(\frac{q_{\mathrm{y}}}{2}\right) A_{\mathrm{xz}}^2\right] \tilde{\chi}_{\mathrm{xx}}(\mathbf{q}) \\ & 8\gamma^2 \sum_{\mathbf{q}} \left[\sin^2\left(\frac{q_{\mathrm{x}}}{2}\right)\sin^2\left(\frac{q_{\mathrm{y}}}{2}\right) A_{\mathrm{xy}}^2 + \cos^2\left(\frac{q_{\mathrm{x}}}{2}\right)\sin^2\left(\frac{q_{\mathrm{y}}}{2}\right) A_{\mathrm{yz}}^2\right] \tilde{\chi}_{\mathrm{yy}}(\mathbf{q}) \\ & 8\gamma^2 \sum_{\mathbf{q}} \left[\sin^2\left(\frac{q_{\mathrm{x}}}{2}\right)\cos^2\left(\frac{q_{\mathrm{y}}}{2}\right) A_{\mathrm{xz}}^2 + \cos^2\left(\frac{q_{\mathrm{x}}}{2}\right)\cos^2\left(\frac{q_{\mathrm{y}}}{2}\right) A_{\mathrm{zz}}^2\right] \tilde{\chi}_{\mathrm{zz}}(\mathbf{q}) \end{aligned} \tag{9}$$

and:

$$\begin{aligned}\left(\frac{1}{T_1 T}\right)_{\mathrm{z}} = & 8\gamma^2 \sum_{\mathbf{q}} \left[\cos^2\left(\frac{q_{\mathrm{x}}}{2}\right)\cos^2\left(\frac{q_{\mathrm{y}}}{2}\right) A_{\mathrm{xx}}^2 + \sin^2\left(\frac{q_{\mathrm{x}}}{2}\right)\sin^2\left(\frac{q_{\mathrm{y}}}{2}\right) A_{\mathrm{xy}}^2\right] \tilde{\chi}_{\mathrm{xx}}(\mathbf{q}) \\ & 8\gamma^2 \sum_{\mathbf{q}} \left[\cos^2\left(\frac{q_{\mathrm{x}}}{2}\right)\cos^2\left(\frac{q_{\mathrm{y}}}{2}\right) A_{\mathrm{yy}}^2 + \sin^2\left(\frac{q_{\mathrm{x}}}{2}\right)\sin^2\left(\frac{q_{\mathrm{y}}}{2}\right) A_{\mathrm{xy}}^2\right] \tilde{\chi}_{\mathrm{yy}}(\mathbf{q}) \\ & 8\gamma^2 \sum_{\mathbf{q}} \left[\sin^2\left(\frac{q_{\mathrm{x}}}{2}\right)\cos^2\left(\frac{q_{\mathrm{y}}}{2}\right) A_{\mathrm{xz}}^2 + \cos^2\left(\frac{q_{\mathrm{x}}}{2}\right)\sin^2\left(\frac{q_{\mathrm{y}}}{2}\right) A_{\mathrm{yz}}^2\right] \tilde{\chi}_{\mathrm{zz}}(\mathbf{q}) \end{aligned} \tag{10}$$

If we approximate the magnetic susceptibility as delta-functions peaked at the magnetic ordering vectors $\mathbf{Q}_1 = (\pi, 0)$ and $\mathbf{Q}_2 = (0, \pi)$, we obtain:

$$\left(T_1 T\right)_{\mathrm{x}}^{-1} = \frac{8\gamma^2 A_{\mathrm{xz}}^2}{\Gamma}\left[\chi_{\mathrm{xx}}^2(\mathbf{Q}_1) + \chi_{\mathrm{yy}}^2(\mathbf{Q}_2) + \chi_{\mathrm{zz}}^2(\mathbf{Q}_2)\right] \tag{11}$$

$$\left(T_1 T\right)_{\mathrm{y}}^{-1} = \frac{8\gamma^2 A_{\mathrm{xz}}^2}{\Gamma}\left[\chi_{\mathrm{xx}}^2(\mathbf{Q}_1) + \chi_{\mathrm{yy}}^2(\mathbf{Q}_2) + \chi_{\mathrm{zz}}^2(\mathbf{Q}_1)\right] \tag{12}$$

$$\left(T_1 T\right)_{\mathrm{z}}^{-1} = \frac{8\gamma^2 A_{\mathrm{xz}}^2}{\Gamma}\left[\chi_{\mathrm{zz}}^2(\mathbf{Q}_1) + \chi_{\mathrm{zz}}^2(\mathbf{Q}_2)\right] \tag{13}$$

These equations can be inverted to extract the quantities:

$$\chi^2_{\mathrm{zz}}(\mathbf{Q}_1) = \frac{\Gamma}{16\gamma^2 A^2_{\mathrm{xz}}}\left[-(T_1T)^{-1}_{\mathrm{y}}(-\varepsilon) + (T_1T)^{-1}_{\mathrm{y}}(\varepsilon) + (T_1T)^{-1}_{\mathrm{z}}(\varepsilon)\right] \tag{14}$$

$$\chi^2_{\mathrm{zz}}(\mathbf{Q}_2) = \frac{\Gamma}{16\gamma^2 A^2_{\mathrm{xz}}}\left[(T_1T)^{-1}_{\mathrm{y}}(-\varepsilon) - (T_1T)^{-1}_{\mathrm{y}}(\varepsilon) + (T_1T)^{-1}_{\mathrm{z}}(\varepsilon)\right] \tag{15}$$

$$\chi^2_{\mathrm{xx}}(\mathbf{Q}_1) + \chi^2_{\mathrm{yy}}(\mathbf{Q}_2) = \frac{\Gamma}{16\gamma^2 A^2_{\mathrm{xz}}}\left[(T_1T)^{-1}_{\mathrm{y}}(-\varepsilon) + (T_1T)^{-1}_{\mathrm{y}}(\varepsilon) - (T_1T)^{-1}_{\mathrm{z}}(\varepsilon)\right], \tag{16}$$

using the fact that $(T_1T)^{-1}_{\mathrm{x}}(\varepsilon) = (T_1T)^{-1}_{\mathrm{y}}(-\varepsilon)$. These quantities are plotted in Fig. 2(e) of the main text.

Although useful for a qualitative analysis, this approximation neglects the important fact that the magnetic fluctuations have finite correlation lengths ξ. To model this effect, we consider susceptibilities peaked at $\mathbf{Q}_1$ and $\mathbf{Q}_2$, as seen by neutron scattering experiments (the amplitude χ_0 of the susceptibilities is absorbed in Γ, for convenience) [14]:

$$\begin{aligned}
\Gamma\tilde{\chi}_{\mathrm{xx}}(\mathbf{q}) &= \frac{1}{\left[(\xi_{\mathrm{x}}^{-2} - \varphi_{\mathrm{xy}}) + (\cos q_{\mathrm{x}} - \cos q_{\mathrm{y}} + 2)\right]^2} + \frac{1}{\left[\left(\xi_{\mathrm{y}}^{-2} + \varphi_{\mathrm{yx}}\right) + (-\cos q_{\mathrm{x}} + \cos q_{\mathrm{y}} + 2)\right]^2}\\
\Gamma\tilde{\chi}_{\mathrm{yy}}(\mathbf{q}) &= \frac{1}{\left[\left(\xi_{\mathrm{y}}^{-2} - \varphi_{\mathrm{yx}}\right) + (\cos q_{\mathrm{x}} - \cos q_{\mathrm{y}} + 2)\right]^2} + \frac{1}{\left[(\xi_{\mathrm{x}}^{-2} + \varphi_{\mathrm{xy}}) + (-\cos q_{\mathrm{x}} + \cos q_{\mathrm{y}} + 2)\right]^2}\\
\Gamma\tilde{\chi}_{\mathrm{zz}}(\mathbf{q}) &= \frac{1}{\left[(\xi_{\mathrm{z}}^{-2} - \varphi_{\mathrm{zz}}) + (\cos q_{\mathrm{x}} - \cos q_{\mathrm{y}} + 2)\right]^2} + \frac{1}{\left[(\xi_{\mathrm{z}}^{-2} + \varphi_{\mathrm{zz}}) + (-\cos q_{\mathrm{x}} + \cos q_{\mathrm{y}} + 2)\right]^2}.
\end{aligned} \tag{17}$$

Note that we have three different correlation lengths: ξ_{x} corresponds to in-plane spin fluctuations with spins parallel to the ordering vector direction; ξ_{y} corresponds to in-plane spin fluctuations with spins perpendicular to the ordering vector direction; and ξ_{z} corresponds to out-of-plane spin fluctuations. This spin anisotropy originates from the spin-orbit coupling, as shown in Ref. [4]. The nematic order parameters $\varphi_{\alpha\beta}$ split the tetragonal degeneracy between $\chi_{\mathrm{xx}}(\mathbf{Q}_1)$ and $\chi_{\mathrm{yy}}(\mathbf{Q}_2)$, between $\chi_{\mathrm{xx}}(\mathbf{Q}_2)$ and $\chi_{\mathrm{yy}}(\mathbf{Q}_1)$, and between $\chi_{\mathrm{zz}}(\mathbf{Q}_1)$ and $\chi_{\mathrm{zz}}(\mathbf{Q}_2)$. They are related to the external strain ε according to the nematic susceptibilities $\chi^{\mathrm{nem}}_{\alpha\beta}$, i.e. $\varphi_{\alpha\beta} = \chi^{\mathrm{nem}}_{\alpha\beta}\varepsilon$.

Substituting these expressions in Eqs. (8), (9), and (10) give:

$$\frac{\Gamma}{8\gamma^2}\left(\frac{1}{T_1 T}\right)_{\mathrm{x}} = A_{\mathrm{xy}}^2\left[J_1\left(\xi_{\mathrm{x}}^{-2}-\varphi_{\mathrm{xy}}\right)+J_1\left(\xi_{\mathrm{y}}^{-2}+\varphi_{\mathrm{yx}}\right)\right]+A_{\mathrm{xz}}^2\left[J_3\left(\xi_{\mathrm{x}}^{-2}-\varphi_{\mathrm{xy}}\right)+J_2\left(\xi_{\mathrm{y}}^{-2}+\varphi_{\mathrm{yx}}\right)\right]$$
$$+A_{\mathrm{yy}}^2\left[J_1\left(\xi_{\mathrm{y}}^{-2}-\varphi_{\mathrm{yx}}\right)+J_1\left(\xi_{\mathrm{x}}^{-2}+\varphi_{\mathrm{xy}}\right)\right]+A_{\mathrm{yz}}^2\left[J_2\left(\xi_{\mathrm{y}}^{-2}-\varphi_{\mathrm{yx}}\right)+J_3\left(\xi_{\mathrm{x}}^{-2}+\varphi_{\mathrm{xy}}\right)\right]$$
$$+A_{\mathrm{yz}}^2\left[J_2\left(\xi_{\mathrm{z}}^{-2}-\varphi_{\mathrm{zz}}\right)+J_3\left(\xi_{\mathrm{z}}^{-2}+\varphi_{\mathrm{zz}}\right)\right]+A_{\mathrm{zz}}^2\left[J_1\left(\xi_{\mathrm{z}}^{-2}-\varphi_{\mathrm{zz}}\right)+J_1\left(\xi_{\mathrm{z}}^{-2}+\varphi_{\mathrm{zz}}\right)\right] \tag{18}$$

as well as

$$\frac{\Gamma}{8\gamma^2}\left(\frac{1}{T_1 T}\right)_{\mathrm{y}} = A_{\mathrm{xx}}^2\left[J_1\left(\xi_{\mathrm{x}}^{-2}-\varphi_{\mathrm{xy}}\right)+J_1\left(\xi_{\mathrm{y}}^{-2}+\varphi_{\mathrm{yx}}\right)\right]+A_{\mathrm{xz}}^2\left[J_3\left(\xi_{\mathrm{x}}^{-2}-\varphi_{\mathrm{xy}}\right)+J_2\left(\xi_{\mathrm{y}}^{-2}+\varphi_{\mathrm{yx}}\right)\right]$$
$$+A_{\mathrm{xy}}^2\left[J_1\left(\xi_{\mathrm{y}}^{-2}-\varphi_{\mathrm{yx}}\right)+J_1\left(\xi_{\mathrm{x}}^{-2}+\varphi_{\mathrm{xy}}\right)\right]+A_{\mathrm{yz}}^2\left[J_2\left(\xi_{\mathrm{y}}^{-2}-\varphi_{\mathrm{yx}}\right)+J_3\left(\xi_{\mathrm{x}}^{-2}+\varphi_{\mathrm{xy}}\right)\right]$$
$$+A_{\mathrm{xz}}^2\left[J_3\left(\xi_{\mathrm{z}}^{-2}-\varphi_{\mathrm{zz}}\right)+J_2\left(\xi_{\mathrm{z}}^{-2}+\varphi_{\mathrm{zz}}\right)\right]+A_{\mathrm{zz}}^2\left[J_1\left(\xi_{\mathrm{z}}^{-2}-\varphi_{\mathrm{zz}}\right)+J_1\left(\xi_{\mathrm{z}}^{-2}+\varphi_{\mathrm{zz}}\right)\right], \tag{19}$$

and

$$\frac{\Gamma}{8\gamma^2}\left(\frac{1}{T_1 T}\right)_{\mathrm{z}} = A_{\mathrm{xx}}^2\left[J_1\left(\xi_{\mathrm{x}}^{-2}-\varphi_{\mathrm{xy}}\right)+J_1\left(\xi_{\mathrm{y}}^{-2}+\varphi_{\mathrm{yx}}\right)\right]+A_{\mathrm{xy}}^2\left[J_1\left(\xi_{\mathrm{x}}^{-2}-\varphi_{\mathrm{xy}}\right)+J_1\left(\xi_{\mathrm{y}}^{-2}+\varphi_{\mathrm{yx}}\right)\right]$$
$$+A_{\mathrm{yy}}^2\left[J_1\left(\xi_{\mathrm{y}}^{-2}-\varphi_{\mathrm{yx}}\right)+J_1\left(\xi_{\mathrm{x}}^{-2}+\varphi_{\mathrm{xy}}\right)\right]+A_{\mathrm{xy}}^2\left[J_1\left(\xi_{\mathrm{y}}^{-2}-\varphi_{\mathrm{yx}}\right)+J_1\left(\xi_{\mathrm{x}}^{-2}+\varphi_{\mathrm{xy}}\right)\right]$$
$$+A_{\mathrm{xz}}^2\left[J_3\left(\xi_{\mathrm{z}}^{-2}-\varphi_{\mathrm{zz}}\right)+J_2\left(\xi_{\mathrm{z}}^{-2}+\varphi_{\mathrm{zz}}\right)\right]+A_{\mathrm{yz}}^2\left[J_2\left(\xi_{\mathrm{z}}^{-2}-\varphi_{\mathrm{zz}}\right)+J_3\left(\xi_{\mathrm{z}}^{-2}+\varphi_{\mathrm{zz}}\right)\right]. \tag{20}$$

Here, we defined the integrals:

$$J_1(r)=\int_{-\pi}^{\pi}\int_{-\pi}^{\pi}\frac{dq_{\mathrm{x}}dq_{\mathrm{y}}}{(2\pi)^2}\frac{\cos^2\left(\frac{q_{\mathrm{x}}}{2}\right)\cos^2\left(\frac{q_{\mathrm{y}}}{2}\right)}{\left[r+(\cos q_{\mathrm{x}}-\cos q_{\mathrm{y}}+2)\right]^2}\equiv\int_{-\pi}^{\pi}\int_{-\pi}^{\pi}\frac{dq_{\mathrm{x}}dq_{\mathrm{y}}}{(2\pi)^2}\frac{\sin^2\left(\frac{q_{\mathrm{x}}}{2}\right)\sin^2\left(\frac{q_{\mathrm{y}}}{2}\right)}{\left[r+(\cos q_{\mathrm{x}}-\cos q_{\mathrm{y}}+2)\right]^2}$$
$$J_2(r)=\int_{-\pi}^{\pi}\int_{-\pi}^{\pi}\frac{dq_{\mathrm{x}}dq_{\mathrm{y}}}{(2\pi)^2}\frac{\cos^2\left(\frac{q_{\mathrm{x}}}{2}\right)\sin^2\left(\frac{q_{\mathrm{y}}}{2}\right)}{\left[r+(\cos q_{\mathrm{x}}-\cos q_{\mathrm{y}}+2)\right]^2}$$
$$J_3(r)=\int_{-\pi}^{\pi}\int_{-\pi}^{\pi}\frac{dq_{\mathrm{x}}dq_{\mathrm{y}}}{(2\pi)^2}\frac{\sin^2\left(\frac{q_{\mathrm{x}}}{2}\right)\cos^2\left(\frac{q_{\mathrm{y}}}{2}\right)}{\left[r+(\cos q_{\mathrm{x}}-\cos q_{\mathrm{y}}+2)\right]^2}. \tag{21}$$

In the limit $\xi_i^{-2}\pm\varphi_{\alpha\beta}\ll 1$, we can approximate the integrals by expanding the integrand near $(\pi,0)$, yielding:

$$
\begin{aligned}
J_1(r) &\approx \frac{1}{4\pi}\ln\left(\frac{\Lambda_1}{\sqrt{r}}\right)\\
J_2(r) &\approx \frac{1}{8\pi}\left[1-\frac{r}{2}\ln\left(\frac{\Lambda_2}{\sqrt{r}}\right)\right]\\
J_3(r) &\approx \frac{1}{2\pi r}
\end{aligned}
\tag{22}
$$

where $\Lambda_1 \approx 1.45$ and $\Lambda_2 \approx 3.2$ for $r < 0.5$, according to numerical evaluations of the integrals. Note that, as expected from symmetry considerations, $(T_1T)^{-1}_{\mathrm{x}}(-\varepsilon) = (T_1T)^{-1}_{\mathrm{y}}(\varepsilon)$ and $(T_1T)^{-1}_{\mathrm{z}}(-\varepsilon) = (T_1T)^{-1}_{\mathrm{z}}(\varepsilon)$.

Supplementary Note 4. FITTING THE SPIN-LATTICE RELAXATION RATE DATA

The expressions for $(T_1T)^{-1}_{\alpha}$ given above depend on six parameters: ξ_{x}, ξ_{y}, ξ_{z}, φ_{xy}, φ_{yx}, and φ_{zz}. We first fit the zero-strain data shown in Figs. 2(b) and 2(d) of the main text assuming all the $\varphi_{\alpha\beta} = 0$, and that $\xi_{\mathrm{y}} = \xi_{\mathrm{x}}$. Because the Landau damping term, Γ, is unknown, one cannot simply extract the $\xi_{x,\mathrm{z}}$ directly from the data. However, the ratio of $(T_1T)^{-1}_{\mathrm{x}}/(T_1T)^{-1}_{\mathrm{z}}$ does constrain the data and enable us to fit the data using the temperature-dependent correlation lengths shown in Fig. 4(a) of the main text. The hyperfine coupling constants are given by: $A_{\mathrm{xx}} = A_{\mathrm{yy}} = 0.66$ T/μ_B, $A_{\mathrm{zz}} = 0.47$ T/μ_B, and $A_{\mathrm{xz}} = A_{\mathrm{yz}} = 0.43$ T/μ_B [1], and we assume the value $A_{\mathrm{xy}} = 0.33$ T/μ_B [13].

Using these values for $\xi_{x,\mathrm{z}}$ and assuming that $\xi_{\mathrm{y}} = \xi_{\mathrm{x}}$, we then proceed to fit the strain-dependent $(T_1T)^{-1}$ data to the three nematic order parameters, $\varphi_{\mathrm{xy}} = \chi^{\mathrm{nem}}_{\mathrm{xy}}\varepsilon$, $\varphi_{\mathrm{yx}} = \chi^{\mathrm{nem}}_{\mathrm{yx}}\varepsilon$, and $\varphi_{\mathrm{zz}} = \chi^{\mathrm{nem}}_{\mathrm{zz}}\varepsilon$, where the $\chi^{\mathrm{nem}}_{\alpha\beta}$ are the static nematic susceptibilities of the three components of the nematic order. These data are shown in Fig. 4(b) of the main text as a function of temperature.

Supplementary Note 5. SAMPLE CHARACTERIZATION AND RESISTIVITY ANISOTROPY

In Supplementary Figure 2 we plot the resistivity of the two samples used in this study (in $H \parallel a$ and $H \parallel c$ configurations). The samples had residual resistivity ratios (rrr) of 7.1 and

6.2, respectively, with very similar $\rho(T)$ temperature dependences. For reference we show the temperature-dependent resistivity of as-grown samples studied by Tanatar *et al.* [2] with residual resistivity ratio ∼3, and long-term annealed samples with residual resistivity ratio of ∼30 [3]. Reducing residual resistivity in the samples leads to an increase of the structural transition temperature, as shown in inset in the left panel. For the two samples studied T_s was 136.7 K ($H \parallel a$) and 135.6 K ($H \parallel c$, intermediate between low and high residual resistivity ratio samples.

In the right panel of Supplementary Figure 2 we plot the difference of the principal components of the in-plane resistivity in the orthorhombic phase, $\rho_b - \rho_a$, for low (green line, rrr∼3, Ref. 2) and high (blue line, rrr∼30, Ref. 3) residual resistivity ratio samples. While in the former a big anisotropy is found in the $T = 0$ limit, negligible anisotropy is found in the later. The samples used in this study show negligible difference in $T = 0$ limit, similar to high quality samples.

This sample characterization suggests that the samples used in this study do not reveal extrinsic high anisotropy in $T = 0$ limit. They are representative of high quality annealed samples. Comparisons of the difference curves for annealed samples [3] and samples of our study reveal comparable differences in magnitude at the maximum immediately below T_s, supporting this conclusion.

Supplementary Note 6. ENHANCEMENT OF T_N UNDER STRAIN

Supplementary Figure 3 shows T_N versus strain for three data points based on our observations of the NMR spectra for $\mathbf{H}_0 \perp \mathbf{c}$. When the system orders antiferromagnetically, a static internal field develops along the **c** axis that shifts the resonance frequency. The strain and temperature values shown in the figure represent the points where we observe shifts of the spectrum consistent with the presence of such a field. This behavior arises because the large nematic susceptibility increases the correlation length, ξ, which gives rise to a non-linear effect in which T_N is enhanced by strain. In fact, one case estimate the the strain dependence of T_N because χ diverges when $\xi_x^{-2}(T) - \chi_{xy}^{nem}(T)\varepsilon = 0$. Using the fitted parameters for ξ_x and χ_{xy}^{nem} we estimate T_N versus ε, and the result agrees well with the measured values. These results agree with previous neutron scattering studies [15].

SUPPLEMENTARY REFERENCES

[1] Kentaro Kitagawa, Naoyuki Katayama, Kenya Ohgushi, Makoto Yoshida, and Masashi Takigawa, Commensurate itinerant antiferromagnetism in $BaFe_2As_2$: ^{75}As-NMR studies on a self-flux grown single crystal, J. Phys. Soc. Jpn. **77**, 114709 (2008).

[2] M. A. Tanatar, E. C. Blomberg, A. Kreyssig, M. G. Kim, N. Ni, A. Thaler, S. L. Bud'ko, P. C. Canfield, A. I. Goldman, I. I. Mazin, and R. Prozorov, Uniaxial-strain mechanical detwinning of $CaFe_2As_2$ and $BaFe_2As_2$ crystals: Optical and transport study, Phys. Rev. B **81**, 184508 (2010).

[3] S. Ishida, T. Liang, M. Nakajima, K. Kihou, C. H. Lee, A. Iyo, H. Eisaki, T. Kakeshita, T. Kida, M. Hagiwara, Y. Tomioka, T. Ito, and S. Uchida, Manifestations of multiple-carrier charge transport in the magnetostructurally ordered phase of $BaFe_2As_2$, Phys. Rev. B **84**, 184514 (2011).

[4] Morten H. Christensen, Jian Kang, Brian M. Andersen, Ilya Eremin, and Rafael M. Fernandes, Spin reorientation driven by the interplay between spin-orbit coupling and hund's rule coupling in iron pnictides, Phys. Rev. B **92**, 214509 (2015).

[5] A. P. Dioguardi, T. Kissikov, C. H. Lin, K. R. Shirer, M. M. Lawson, H.-J. Grafe, J.-H. Chu, I. R. Fisher, R. M. Fernandes, and N. J. Curro, NMR evidence for inhomogeneous nematic fluctuations in $BaFe_2(As_{1-x}P_x)_2$, Phys. Rev. Lett. **116**, 107202 (2016).

[6] Tetsuya Iye, Marc-Henri Julien, Hadrien Mayaffre, Mladen Horvatić, Claude Berthier, Kenji Ishida, Hiroaki Ikeda, Shigeru Kasahara, Takasada Shibauchi, and Yuji Matsuda, Emergence of orbital nematicity in the tetragonal phase of $BaFe_2(As_{1-x}P_x)_2$, J. Phys. Soc. Jpn. **84**, 043705 (2015).

[7] F. L. Ning, K. Ahilan, T. Imai, A. S. Sefat, R. Jin, M. A. McGuire, B. C. Sales, and D. Mandrus, ^{59}Co and ^{75}As NMR investigation of lightly doped $Ba(Fe_{1-x}Co_x)_2As_2$ ($x = 0.02$, 0.04), Phys. Rev. B **79**, 140506 (2009).

[8] A. P. Dioguardi, N. apRoberts Warren, A. C. Shockley, S. L. Bud'ko, N. Ni, P. C. Canfield, and N. J. Curro, Local magnetic inhomogeneities in $Ba(Fe_{1-x}Ni_x)_2As_2$ as seen via As-75 NMR, Phys. Rev. B **82**, 140411(R) (2010).

[9] Hikaru Takeda, Takashi Imai, Makoto Tachibana, Jonathan Gaudet, Bruce D. Gaulin, Bayrammurad I. Saparov, and Athena S. Sefat, Cu substitution effects on the local magnetic properties of $Ba(Fe_{1-x}Cu_x)_2As_2$: A site-selective ^{75}As and ^{63}Cu NMR study, Phys. Rev. Lett. **113**, 117001 (2014).

[10] S-H. Baek, D. V. Efremov, J. M. Ok, J. S. Kim, Jeroen van den Brink, and B. Büchner, Orbital-driven nematicity in FeSe, Nat. Mater. **14**, 210–214 (2015).

[11] Mingquan He, Liran Wang, Felix Ahn, Frédéric Hardy, Thomas Wolf, Peter Adelmann, Jörg Schmalian, Ilya Eremin, and Christoph Meingast, Dichotomy between in-plane magnetic susceptibility and resistivity anisotropies in extremely strained $BaFe_2As_2$, Nat. Commun. **8**, 504 (2017).

[12] Andrew Smerald and Nic Shannon, Angle-resolved NMR: Quantitative theory of ^{75}As T_1 relaxation rate in $BaFe_2As_2$, Phys. Rev. B **84**, 184437 (2011).

[13] T. Kissikov, A. P. Dioguardi, E. I. Timmons, M. A. Tanatar, R. Prozorov, S. L. Bud'ko, P. C. Canfield, R. M. Fernandes, and N. J. Curro, NMR study of nematic spin fluctuations in a detwinned single crystal of underdoped $Ba(Fe_{2-x}Co_x)_2As_2$, Phys. Rev. B **94**, 165123 (2016).

[14] Xingye Lu, J. T. Park, Rui Zhang, Huiqian Luo, Andriy H. Nevidomskyy, Qimiao Si, and Pengcheng Dai, Nematic spin correlations in the tetragonal state of uniaxial-strained $BaFe_{2-x}Ni_xAs_2$, Science **345**, 657 – 660 (2014).

[15] Chetan Dhital, Tom Hogan, Z. Yamani, Robert J. Birgeneau, W. Tian, M. Matsuda, A. S. Sefat, Ziqiang Wang, and Stephen D. Wilson, Evolution of antiferromagnetic susceptibility under uniaxial pressure in $Ba(Fe_{1-x}Co_x)_2As_2$, Phys. Rev. B **89**, 214404 (2014).
